# Higher Expression of SPP1 Predicts Poorer Survival Outcomes in Head and Neck Cancer

**DOI:** 10.1155/2021/8569575

**Published:** 2021-12-23

**Authors:** Tongwu Bie, Xuewen Zhang

**Affiliations:** Department of Ear-Nose-Throat, Huai'an Second People's Hospital, The Affiliated Huai'an Hospital of Xuzhou Medical University, Huai'an, Jiangsu 223002, China

## Abstract

Secreted phosphoprotein 1 (SPP1) participated in various biological processes in many cancers, including immune response, tumor progression, and prognosis. However, SPP1 in head and neck squamous cell carcinoma (HNSCC) remains unknown. Clinical-genetic data of HNSCC were obtained from The Cancer Genome Atlas (TCGA) database. The differential expression of SPP1 in HNSCC tissues and adjacent normal tissues was quantified by bioinformatics methods and verified by western blot and other differential biological methods. We concluded that SPP1 is significantly upregulated in tumor tissues and can become a prognostic biomarker for HNSCC.

## 1. Introduction

As reported by cancer statistics, head and neck squamous cell carcinoma (HNSCC) annually affects about 550000 people worldwide and ranks the sixth leading cause of cancer-related deaths [1]. The oral cavity, pharynx, and larynx can become origin sites for HNSCC. Because of the lesion location in the upper aerodigestive tract, the treatments for HNSCC usually lower the life quality of patients, such as dyspnea, pararthria, dysphagia, and even disfigurement of appearance [2]. Recent advances and emerging therapies in the clinical management of HNSCC made the 5-year overall survival (OS) 40%-60% in the past decades [3, 4]. The inherent heterogeneity of tumor cells leads to drug resistance, which limits the prognosis of these patients [5]. Thus, the molecular mechanisms and treatment strategies for tumor multidrug resistance have attracted enormous research interests [6].

Secreted phosphoprotein 1 (SPP1), also named as osteopontin (OPN), is a multifunctional marticellular glycoprotein synthesized by multiple cells and tissues and plays a pivotal role in immune response [7] and cancer progression [8]. SPP1 is abnormally elevated positively correlated with the severity of tumor malignancy and chemoresistance in breast cancer [9], non-small-cell lung cancer [10], prostate cancer [11], and liver cancer [12]. SPP1 can bind and activate multiple downstream signaling pathways, which can activate tumor growth and invasion and limit the antitumor function of immune cells [13]. For instance, integrin *α*v*β*3 binding to SPP1 can promote cellular migration through the FAK, ERK1/2, and NF-*κ*B signaling pathways [14] and increase tumor progression and reduce apoptosis of cancer cells via phosphoinositide 3-kinase (PI3K)/Akt/mTOR and JAK2/STAT3 signaling pathways [12, 15]. But the regulation of SPP1 in HNSCC remains unknown, which requires further elucidation.

In this study, we used bioinformatics methods and biological assay to assess the prognostic values of SPP1 in HNSCC and analyze the correlation between survival outcomes and SPP1 expression. Moreover, distinctive genomic features correlated with the expression of SPP1 were also analyzed by using The Cancer Genome Atlas (TCGA) database. The purpose of this study is to provide the evidence on SPP1 as a potential biomarker for HNSCC, which could fill the research gaps in previous studies.

## 2. Materials and Methods

### 2.1. Oncomine Analysis

Oncomine is publicly accessible at https://www.Oncomine.org, which becomes a compendium of more than 20000 cancer transcriptomes for facilitating the genome-wide expression analyses. There were 715 datasets, with 86733 samples, that provided the transcriptional levels of SPP1 in HNSCC cases.

### 2.2. TCGA Data and Samples

We searched for gene expression data (i.e., SPP1 messenger RNA (mRNA) and corresponding clinical characteristics) related to HNSCC patients from TCGA database, which contained 599 HNSCC and 44 normal samples. We evaluated the association with survival outcomes for each gene.

### 2.3. SPP1 Expression Analysis and Survival

The original gene expression data were preprocessed by the Perl programming language, and the SPP1 expression level was extracted using the “limma” package. Data were visualized using the “beeswarm” package. The survival information and the SPP1 expression level were matched. 563 patients who met the criteria were finally included. The SPP1 mRNA expression level was divided into two groups (high- and low-SPP1 expression group) based on the median expression value. The “survival” package was for computing the Kaplan-Meier (KM) survival curve.

### 2.4. Immune Infiltration Database

Details of immunofluorescence staining and immunohistochemistry are described in Supplementary Materials (available here). The relationship between SPP1 copy number alteration (CNA) and immune infiltration level was explored and revealed via the TIMER database (https://cistrome.shinyapps.io/timer/).

### 2.5. LinkedOmics Database Analysis

Analysis of TCGA was conducted in a cancer-associated database (LinkedOmics; http://www.linkedomics.org/login.php). Significant relationship between genes in TCGA-HNSC and SPP1 was discovered. Establishment of a heat map plot for the coexpressed genes was through “*LinkFinder*” in LinkedOmics. GO and KEGG analyses were performed with “clusterProfiler” after obtaining strong coexpressed genes.

### 2.6. GEPIA Database Analysis

Gene Expression Profiling Interactive Analysis (GEPIA) (http://gepia.cancer-pku.cn/) involves tumor or normal samples from TCGA, in which the relationship between SPP1 and several coexpressions was evaluated.

### 2.7. Gene Set Enrichment and Functional Annotation

Gene set enrichment analysis (GSEA) has been successfully applied to interpreting the pathway activated in different biological states. In this study, software “GSEA” (https://www.gsea-msigdb.org/) was utilized to identify the gene up- or downregulation after screening gene set size (min = 5, max = 500) and being ranked by the *t*-score. The datasets of “c2.cp.kegg.v7.1.symbols” (MSigDB database) were used for GSEA analysis. The FDR-corrected *q*-value < 0.25 and *P* value < 0.05 were considered statistically significant. Details of immunofluorescence staining, immunohistochemistry, and western blot analysis are described in Supplementary Materials.

### 2.8. Statistical Analysis

SPSS 24.0 was used to analyze statistics. Patients in TCGA were subgrouped by age according to their cognitive functions. Normality was firstly checked in continuous variables, and they were hereafter exhibited as mean ± SD, while categorical variables were presented as percentages (%). Differences between groups were assessed by the *t*-test, one-way ANOVA, or Kruskal-Wallis test for continuous variables and the chi-square or Fisher test for categorical variables, respectively. The post hoc test was applied after the ANOVA and Kruskal-Wallis tests.

Complementing differential expression analysis, correlation analyses were carried out to compute the strength of interrelationships between clinical traits and gene expression traits. Correlations between m6A regulators were computed by Spearman correlation analyses and visualized by using the “corrplot” package in the R program. Univariate analysis examinations, filtering the meaningful independent variables, followed by multivariate logistic regression were conducted to estimate the association between m6A methylation and MCI and AD.

All statistical *P* values were two-tailed, and *P* < 0.05 was regarded as statistically significant.

## 3. Results

### 3.1. The mRNA Expression of SPP1 in HNSCC

**Figure 1:**
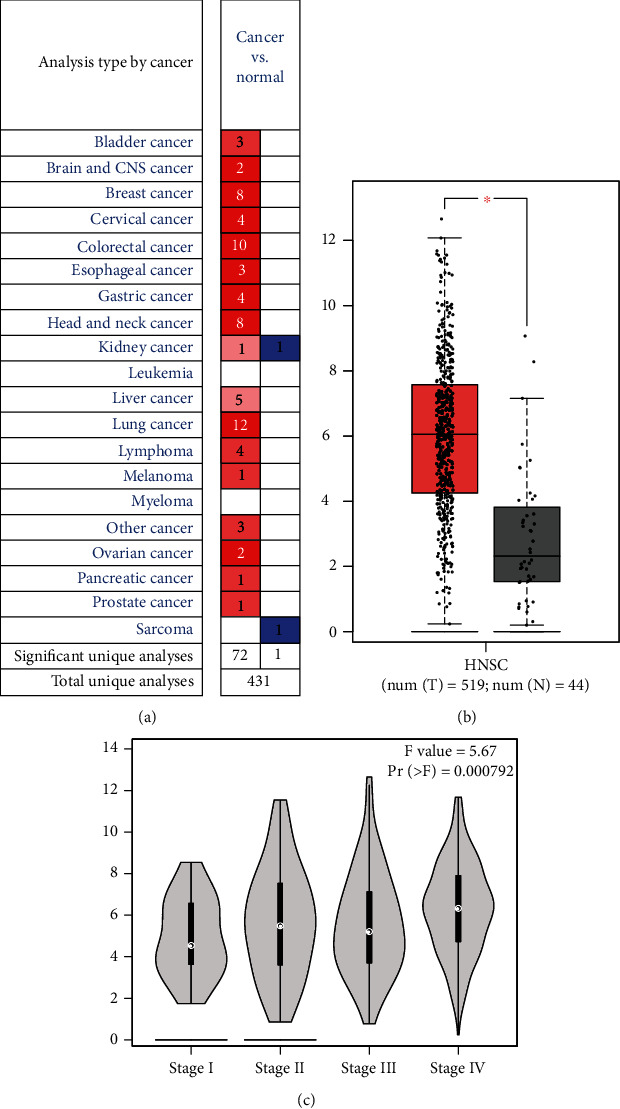
The expression of SPP1 and its association with clinical-pathological variables: (a) Oncomine analysis of the mRNA expression levels of SPP1 in different cancers; (b) comparison of SPP1expression between HNSCC cancer tissues and adjacent normal tissues; (c) the expression of SPP1 grouped by tumor grading.

As shown in Figure 1(a), SPP1 was increased in 8 datasets and none of the datasets showed a reduced level. Then, the difference in SPP1 expression in HNSCC was obtained from TCGA database including 519 HNSCC and 44 adjacent nontumor tissues. The boxplot describes the mRNA expression profiles of SPP1 in HNSCC and adjacent normal tissues.  Figure 1(b) shows that the SPP1 was upregulated in HNSCC tissues compared with adjacent normal tissues (*P* < 0.05). Furthermore, the expression level of SPP1 is exhibited in the pathological stage (Pr = 0.000792, Figure 1(c)).

### 3.2. Verification of SPP1 Upregulation in HNSCC

**Figure 2:**
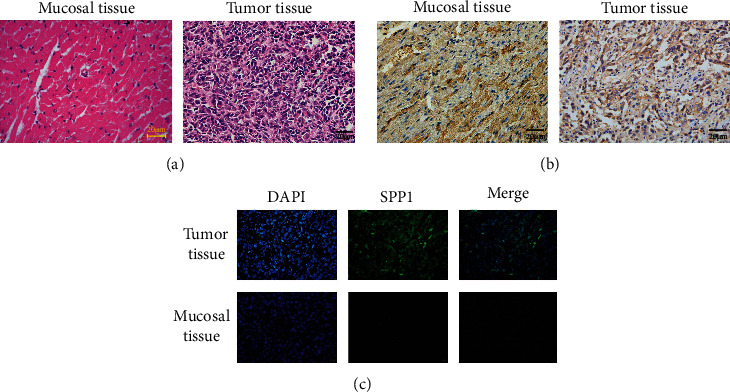
Upregulation of SPP1 in HNSCC: (a) HE in paired HNSCC tissues and their adjacent normal tissues (mucosal tissue); (b) immunochemistry analysis of SPP1 in HNSCC and their adjacent normal tissues; (c) immunofluorescent analysis of SPP1 expression in HNSCC and their adjacent normal tissues.

**Figure 3:**
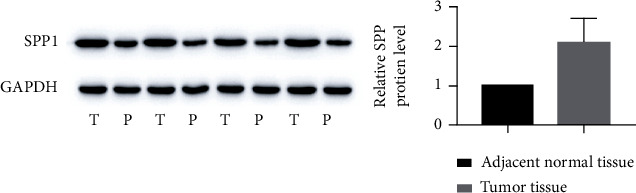
Western blot of tumor and adjacent normal tissues.

To verify the SPP1 expression in HNSCC, we used immunochemistry and immunofluorescence to evaluate the expression of SPP1 in HNSCC tissue of the in vivo xenograft assay. Consistent results were obtained (Figures 2(a)–2(c)). WB results showed that SPP1 was highly expressed in HNSCC tissue compared with adjacent normal tissue in protein level (Figure 3).

### 3.3. Survival Analysis

**Figure 4:**
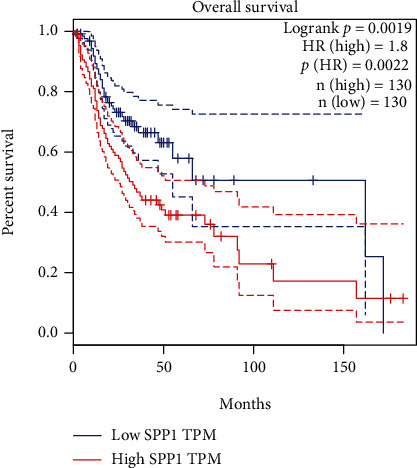
Kaplan-Meier survival estimates of HNSCC patients grouped by expression levels of SPP1.

KM curves revealed that high SPP1 expression indicated higher risk of poor overall survival (Figure 4). The median OS of the high- and low-SPP1 expression group was 32.67 and 58.73 months, respectively. The high expression of 75% patients had a worse survival than the low expression of 25% cases (*P* < 0.0019; Figure 4) under the performance of a tertile analysis.

### 3.4. Verify Coexpression Genes with SPP1 in HNSCC

**Figure 5:**
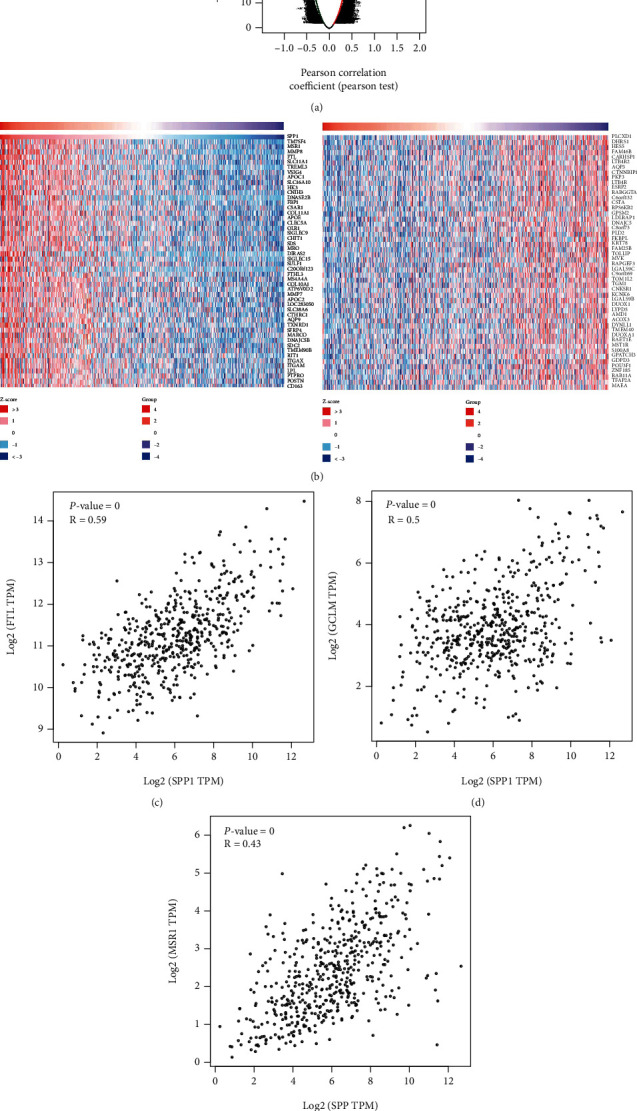
(a) The global PRPF3 highly correlated genes identified by the Pearson test in the HNSCC cohort. (b) Heat maps showing top 50 genes positively and negatively correlated with SPP1 in HNSCC. SPP1 has coexpression with (c) FTL (d) GCLM, and (e) MSR1 in HNSCC.

For evaluating the biological role of SPP1, genes coexpressed with SPP1 in HNSCC were selected. As illustrated in Figure 5(a), 50 genes (marked by red dots) were demonstrated positively associated with SPP1, whereas no genes (blue dots) were found to have a negative correlation with SPP1. Top 50 significant genes are listed in heat maps (Figure 5(b)). In results, FTL, GCLM, and MSR1 has the best corelationship with SPP1 (Figures 5(c)–5(e)), which could be potential signatures in further research.

### 3.5. Signaling Pathways

**Figure 6:**
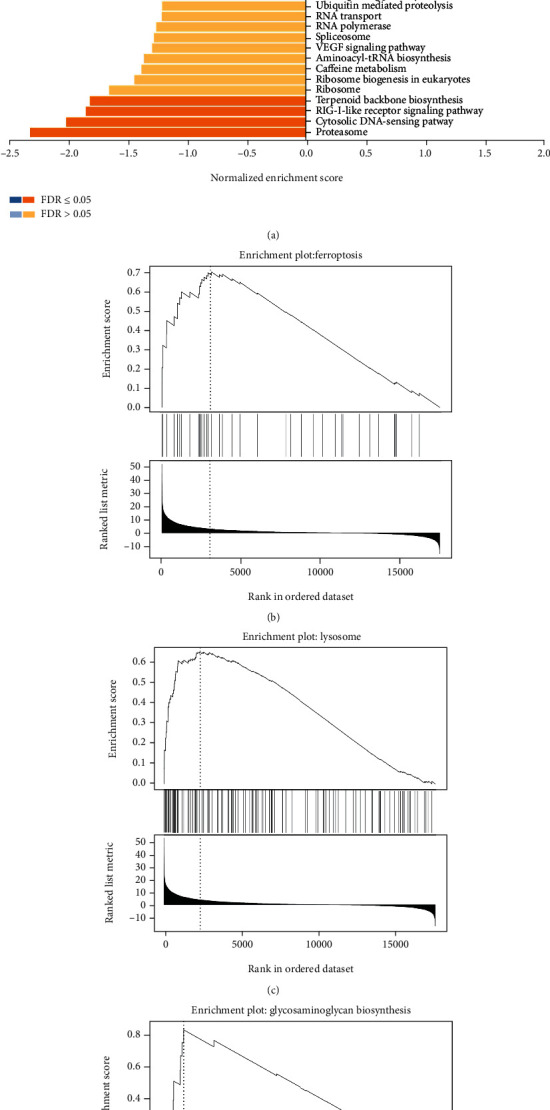
(a) KEGG pathways of SPP1 in the HNSCC cohort in TCGA database. The representative significantly enriched signaling pathways were (b) ferroptosis, (c) lysosome, and (d) glycosaminoglycan biosynthesis.

Based on TCGA, we evaluated the SPP1-related signal pathways via GSEA. Nine signaling pathways including Staphylococcus aureus infection, glycosaminoglycan biosynthesis, lysosome, osteoclast differentiation, protein digestion and absorption, ferroptosis, cholesterol metabolism, glycosaminoglycan biosynthesis_1, and hypertrophic cardiomyopathy (HCM) were differentially enriched in the highly expressed phenotypes of SPP1(FDR < 0.5), whereas 4 signaling pathways involved in proteasome, ribosome, cytosolic DNA-sensing pathway, and terpenoid backbone biosynthesis were enriched in the lower expression of SPP1(Figure 6(a)). Ferroptosis, glycosaminoglycan biosynthesis, and lysosome were verified to have intense relationship with tumor development (Figures 6(b)–6(d)).

### 3.6. Tumor-Infiltrating Lymphocytes

According to the TIMER database, the results indicated that SPP1 induced high immune infiltration of multiple immune cells and can participate in the process of microphage progress.

## 4. Discussion

Several recent studies focused on the expression and predictive value of SPP1 in various cancer cells [11, 16, 17]. In this study, SPP1 was upregulated in HNSCC, and higher SPP1 expression indicated poorer survival.

We investigated the contribution of SPP1 to HNSCC progression. Furthermore, we also found signaling pathways associated with SPP1 in HNSCC to unravel the underlying mechanism of HNSCC progression caused by SPP1. First, we analyzed the RNAseq data and verified that SPP1 mRNA in HNSCC tissues was highly expressed compared with that in adjacent normal tissues. Then, several biological assays were performed for verification. These results indicate that SPP1 might be an oncogene and significantly contribute to the progression of HNSCC. Moreover, SPP1 expressions were different in groups stratified by pathological stages. The expression of SPP1 is positively related to tumor grading. Further analyses showed that SPP1 significantly contributed to tumor differentiation. SPP1 was discovered to be upregulated in undifferentiated tumors in Protein Kinase, DNA-Activated, Catalytic Polypeptide (PRKDC) [18–20]. It was encouraging that SPP1 is related to clinical-pathologic variables at the mRNA level, and PRKDC with increased SPP1 expression could progress to an advanced stage. Additionally, similar conclusions were obtained by several studies at the protein level. Lumican in PRKDC tissues was reported to be higher at the protein level, and further correlations between the lumican protein and tumor grading, OS, and organ and lymph node metastasis status were also found [21–24].

KM curves revealed that the high SPP1 expression means poorer survival outcomes. In brief, SPP1 was a potential biomarker for the OS of HNSCC.

In addition, we discovered multiple gene coexpressions with SPP1 genes in HNSCC by GSEA. Our results reveal that SPP1 might affect these factors for regulating the ferroptosis and lysosome, which has been proven to have effects on head and neck carcinoma.

**Figure 7:**
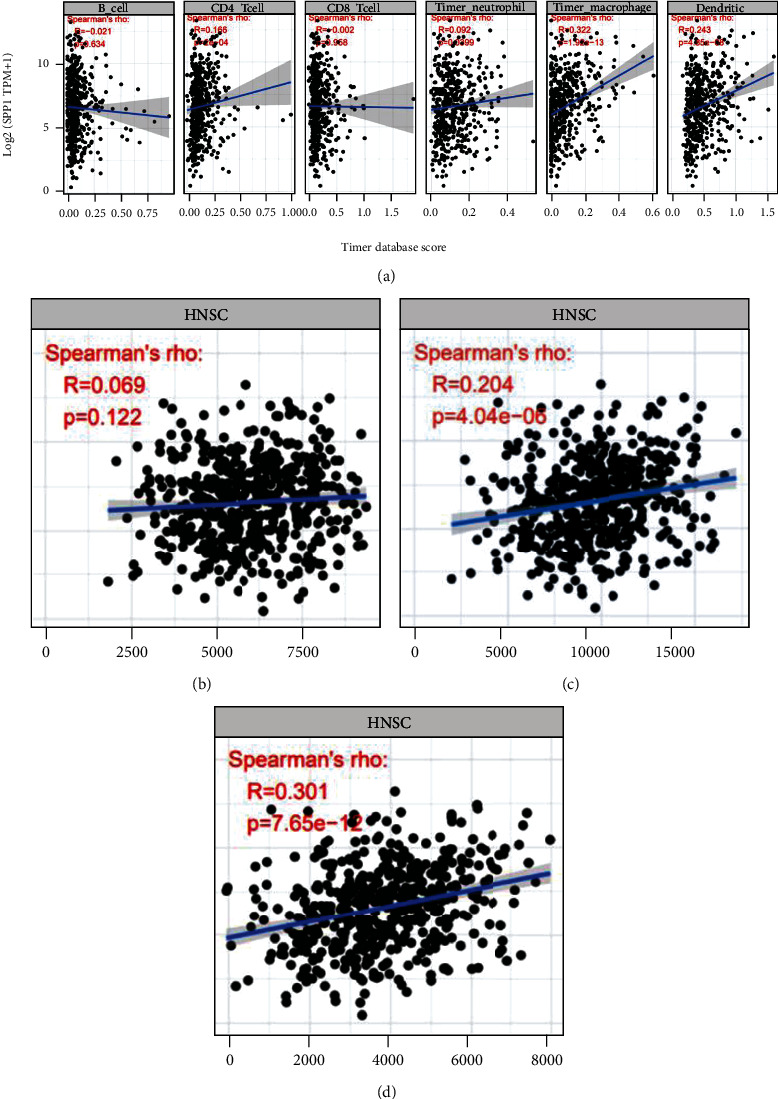
Correlation of SPP1 with immune infiltration level in HNSCC. (a) SPP1 expression is significantly negatively related to tumor purity and has significant positive correlations with infiltrating levels of CD4+ T cells, macrophages, and dendritic cells. Evaluation of the relationship between SPP1 and immune infiltration by the (b) Est_immune score, (c) ESTIMATE score, and (d) stromal score.

Another finding of this study is that SPP1 was found to be associated with diverse immune infiltration levels in HNSCC (Figures 7(b)–7(d)). There is a positive relationship between SPP1 and infiltration level of macrophages and DCs (Figure 7(a)). In addition, the strong correlation between SPP1 and marker genes of immunity indicates the contribution of SPP1 for tumor immunology in HNSCC.

Some limitations in this study should be noted. First, the clinical characteristics are not clear enough and tumor sizes were not provided. Second, treatment details were not provided, which are very important to the survival outcomes of cases. Finally, it is difficult to analyze TCGA database for the protein level and mechanisms of SPP1 in HNSCC.

In conclusion, our research provided the first evidence for the higher expression of SPP1 in HNSCC. The upregulation of SPP1 promotes the occurrence and the progression of HNSCC. Importantly, SPP1 was identified as a biomarker for overall survival in HNSCC, which requires further clinical studies for validation.

## Data Availability

The datasets analyzed during the current study are available from the corresponding authors on reasonable request.

## Supplementary Materials

Supplementary Materials Details of immunofluorescence staining, immunohistochemistry, and western blot analysis are provided.
