# Supplementary material for: Higher Expression of SPP1 Predicts Poorer Survival Outcomes in Head and Neck Cancer
Source: J Immunol Res. 2021 Dec 23;2021:8569575. doi: 10.1155/2021/8569575 (PMC8718292; doi:10.1155/2021/8569575)
Supplement: Supplementary Materials — Details of immunofluorescence staining, immunohistochemistry, and western blot analysis are provided. [file 8569575.f1.docx]

**Higher expression of SPP1 predicts poorer survival outcomes in Head and Neck Cancer**

**Immunofluorescence staining**

Cells were fixed with 4% paraformaldehyde (PFA) for 15 minutes, rinsed with phosphate‐buffered saline (PBS) containing 0.1% Tween‐20, permeabilized with 0.3% Triton X‐100, blocked with 5% bovine serum albumin (BSA), incubated with primary antibodies at 4°C overnight, and were subsequently incubated with fluorescently labeled secondary antibodies at room temperature for 1 hour. The nuclei were stained by using 4′,6‐diamidino‐2‐phenylindole (DAPI) for 4 minutes. All slides were mounted and images were acquired using a Zeiss Axiovert 500 confocal microscope.

**Immunohistochemistry**

Immunohistochemistry (IHC) was performed on paraffin-embedded sections from tumor tissues of nude mice xenografts. Tissue sections were blocked in 10% goat serum supplemented with 0.3% Triton X‐100% and 1% (w/v) BSA for 2 hours at room temperature, and incubated overnight at 4°C with primary antibodies against SPP1 (1:200; Millipore). The sections were incubated with corresponding biotinylated secondary antibodies. Staining was visualized by 3,3’-diaminobenzidine (DAB, Vector Laboratories). The staining intensity was seperately scored: 0, no staining; 1, weak staining (light yellow); 2, moderate staining (yellow brown); 3, strong staining (brown)^[1]^.

**Western blot analysis**

Utilized RIPA lysis buffer with 1% phenylmethanesulfonyl fluoride (PMSF) and DL-dithiothreitol (DTT) to extracted total protein. The BCA protein assay kit (Beyotime Biotechnology) was used to determine the concentration of the protein lysate. Equivalent (30 μg) protein was isolated by 10% SDS-PAGE. Then, the proteins were transferred to PVDF membranes (0.45 mm; Beijing Solarbio Science & Technology Co., China). Before incubated the membranes with SPP1 antibodies (1:1000, R&D Systems, MN, USA) at 4 °C for 12 h, the membranes were blocked at room temperature with 5% BSA for 1 h. Then GAPDH rabbit polyclonal antibody (1:4000, Proteintech, USA) was utilized as a loading control for normalization. HRP-conjugated secondary anti-rabbit antibody (1:4000; ProteinTech Group) was incubated at room temperature about 1 h. Finally, the bands were placed on an Omega Lum G machine (Aplegen, USA) and visualized using ECL reagents (Thermo Fisher Scientific).

**Reference**

1. Meng XM, Zhou Y, Dang T, Tian XY, Kong J. Magnifying chromoendoscopy combined with immunohistochemical staining for early diagnosis of gastric cancer. World J Gastroenterol. 2013 Jan 21;19(3):404-10.
